# Influence of mid-afternoon nap duration and sleep parameters on memory encoding, mood, processing speed, and vigilance

**DOI:** 10.1093/sleep/zsad025

**Published:** 2023-02-13

**Authors:** Ruth L F Leong, TeYang Lau, Andrew R Dicom, Teck Boon Teo, Ju Lynn Ong, Michael W L Chee

**Affiliations:** Department of Medicine, Centre for Sleep and Cognition, Yong Loo Lin School of Medicine, National University of Singapore, Singapore; Department of Medicine, Centre for Sleep and Cognition, Yong Loo Lin School of Medicine, National University of Singapore, Singapore; Department of Medicine, Centre for Sleep and Cognition, Yong Loo Lin School of Medicine, National University of Singapore, Singapore; Department of Medicine, Centre for Sleep and Cognition, Yong Loo Lin School of Medicine, National University of Singapore, Singapore; Department of Medicine, Centre for Sleep and Cognition, Yong Loo Lin School of Medicine, National University of Singapore, Singapore; Department of Medicine, Centre for Sleep and Cognition, Yong Loo Lin School of Medicine, National University of Singapore, Singapore

**Keywords:** nap, memory encoding, mood, processing speed, vigilance, sleep macrostructure

## Abstract

**Study Objectives:**

To determine how mid-afternoon naps of differing durations benefit memory encoding, vigilance, speed of processing (SOP), mood, and sleepiness; to evaluate if these benefits extend past 3 hr post-awakening and to examine how sleep macrostructure during naps modulate these benefits.

**Methods:**

Following short habitual sleep, 32 young adults underwent four experimental conditions in randomized order: wake; naps of 10 min, 30 min, and 60 min duration verified with polysomnography. A 10-min test battery was delivered at a pre-nap baseline, and at 5 min, 30 min, 60 min, and 240 min post-nap. Participants encoded pictures 90 min post-nap and were tested for recognition 210 min later.

**Results:**

Naps ranging from 10 to 60 min increased positive mood and alleviated self-reported sleepiness up to 240 min post-nap. Compared to waking, only naps of 30 min improved memory encoding. Improvements in vigilance were moderate, and benefits for SOP were not observed. Sleep inertia was observed for the 30 min to 60 min naps but was resolved within 30 min after waking. We found no significant associations between sleep macrostructure and memory benefits.

**Conclusions:**

With short habitual sleep, naps ranging from 10 to 60 min had clear and lasting benefits for positive mood and self-reported sleepiness/alertness. Cognitive improvements were moderate, with only the 30 min nap showing benefits for memory encoding. While there is no clear “winning” nap duration, a 30 min nap appears to have the best trade-off between practicability and benefit.

**Clinical Trial ID:**

Effects of Varying Duration of Naps on Cognitive Performance and Memory Encoding, https://www.clinicaltrials.gov/ct2/show/NCT04984824, NCT04984824.

## Graphical Abstract

**Graphical Abstract:**
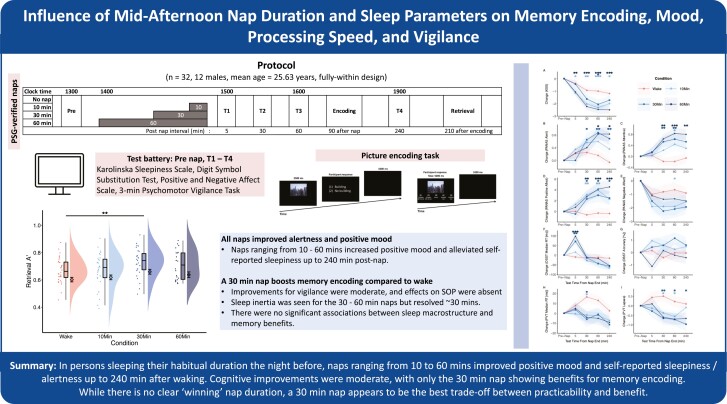

**Statement of Significance**

We evaluated the cognitive benefits of PSG (Polysomnography)-verified, nap durations of 10 min, 30 min, and 60 min for 5 to 240 min after awakening. Naps of any of these durations boosted positive mood and reduced self-reported sleepiness. Compared to wake, only naps of 30 min significantly benefitted recognition at 210 min post encoding. Improvements in speed of processing and vigilance showed less consistent patterns. Sleep macrostructure and memory benefits were not significantly associated. 30 min appears to be the duration to recommend for a mid-afternoon nap while provisioning ~10 min to fall asleep.

## Introduction

Given the prevalence of sleep curtailment in modern society, scheduled napping is increasingly gaining traction as a remedy to counter the detrimental cognitive effects of chronic short nocturnal sleep [1]. Studies have shown that afternoon naps scheduled to coincide with a period of higher sleep propensity [2] can reduce homeostatic sleep pressure [3, 4] and improve vigilance and memory [5–7]. However, pressure to participate in other activities vies with napping for this purpose [8]. Hence, studies comparing the effects of shorter naps (≤30 min) with longer naps (≥60 min) are valuable in informing recommendations on a nap length that balances practicability with meaningful improvements in mood, alertness, and cognitive performance. Specifically, objective measures of sleep obtained during the naps are needed to compare actual sleep obtained rather than the amount of time provisioned for a nap. This will have implications for recommendations on how long one should apportion for a nap.

Previous work comparing different nap durations has mainly examined vigilance and speed of processing, and memory consolidation rather than memory encoding. These studies found that while very brief naps ranging from 30 sec to 5 min show low efficacy, naps of at least 10 min produce measurable benefits on tests of objective alertness [9–11] and processing speed [9, 12, 13]. Memory consolidation is often associated with naps of 60 min or more [14]. However, short naps of 6–10 min [15, 16] and 20–30 min [17, 18] have also been shown to benefit consolidation of declarative and procedural memory. Longer naps might be better for memory consolidation but could negatively affect nocturnal sleep [19], and studies have shown that memory gains depend on the amount of N2, N3, as well as rapid eye movement (REM) obtained during the nap [20–22]. Compared to memory consolidation, few studies have evaluated if nap length influences memory encoding, and its links to learning potential merit study.

Three other variables are important in evaluating the utility of naps: sleep inertia on waking, and how sustained the cognitive and affective benefits are. Sleep inertia refers to the period immediately following wake in which a person’s arousal is temporarily lowered and performance may suffer [23]. While one may avoid sleep inertia and experience immediate improvements in alertness and performance with shorter 10 min naps [9, 12], the benefits of longer naps may be offset [24] by greater sleep inertia [25]. Comparing the temporal evolution of nap benefits would gain from using longer post-nap test intervals. Presently, studies titrating the duration of naps to examine the time course of cognitive benefits have neither examined naps beyond 30 min nor evaluated benefits beyond 185 min. None have assessed how different nap lengths benefit memory encoding.

We sought to address these gaps in a fully within-participant design comparing polysomnographically monitored nap total sleep times of 10 min, 30 min, and 60 min with a wake control condition. Participants encoded pictures 90 min after waking and were tested on their memory 210 min later. Measures of self-reported alertness, mood, sustained vigilance, and speed of processing was serially evaluated at post-nap intervals of 5 min (to assess sleep inertia), 30 min, 60 min, and 240 min.

## Methods

### Participants

A total of 32 participants (12 males, mean age = 25.63 ± 4.33 years, Table 1) were recruited between 2020 and 2022 for the present study. Participants were eligible if they were between 21 and 35 years of age, had no known health or sleep disorders, were not on long-term medication, consumed less than five cups of any type of caffeinated beverages a day, were not shift workers and had not traveled across more than two time zones in the past month. Rather than restricting the sleep of participants prior to the experiment, we specifically selected participants who had habitual short nocturnal sleep of 6–6.5 hr TIB (Time in bed) and slept between 23:00 and 08:00.

**Table 1.** Demographic variables for the sample (*n* = 32) and sleep parameters in means (SD) for the 10 min, 30 min, and 60 min nap conditions

Demographic variables				
Age (years)	25.63 (4.33)	—	—	—
Gender (number of males)	12	—	—	—
Habitual Bedtime (hh:mm)	01:38 (01:06)	—	—	—
Habitual Wake time (hh:mm)	07:25 (01:01)	—	—	—
Habitual TIB (Time in bed) (min)	379.52 (28.83)	—	—	—
Habitual TST (Total sleep time) (min)	314.78 (36.63)	—	—	—
Habitual napper^*^ (%)	56.25%	—	—	—
**Nap variables**	***10 min nap***	***30 min nap***	***60 min nap***	***P***
Time in bed (min)	24.53 (9.76)	43.82 (9.10)	76.26 (10.57)	<.001^†^^,^^‡^^,^^§^
Total sleep time (min)	11.53 (1.64)	31.68 (1.72)	61.88 (3.41)	<.001^†^^,^^‡^^,^^§^
N1 sleep latency (min)	10.28 (7.03)	7.70 (4.86)	7.90 (5.94)	.16
N2 sleep latency (min)	15.52 (8.99)	13.52 (7.21)	14.66 (9.23)	.64
Wake after sleep onset (min)	2.72 (5.75)	4.45 (6.72)	6.48 (7.28)	.11
Sleep efficiency (%)	52.58 (15.95)	74.70 (12.88)	82.43 (10.58)	<.001^†^^,^^§^
N1 sleep (min)	5.33 (3.13)	8.30 (6.76)	10.00 (7.16)	0.003^§^
N2 sleep (min)	5.70 (3.14)	14.57 (4.86)	23.00 (9.09)	<.001^†^^,^^‡^^,^^§^
N3 sleep (min)	0.31 (0.99)	8.52 (7.01)	21.57 (12.77)	<.001^†^^,^^‡^^,^^§^
NREM sleep (min)	11.34 (1.85)	31.39 (1.89)	54.57 (8.75)	<.001^†^^,^^‡^^,^^§^
REM sleep (min)	0.19 (1.06)	0.29 (1.51)	7.31 (8.96)	<.001^‡^^,^^§^
N1 sleep (%)	46.77 (28.06)	25.71 (19.74)	16.12 (11.63)	<.001^†^^,^^§^
N2 sleep (%)	49.15 (26.40)	46.02 (14.84)	37.09 (14.14)	.06
N3 sleep (%)	2.57 (8.22)	27.45 (22.63)	35.12 (21.16)	<.001^†^^,^^§^
REM sleep (%)	1.50 (8.49)	0.83 (4.38)	11.68 (14.26)	<.001^‡^^,^^§^

^*^Reporting napping at least once a week.

^†^ Denotes a significant contrast between the 10 min and 30 min nap conditions.

^‡^ Denotes a significant contrast between the 30 min and 60 min nap conditions.

^§^ Denotes a significant contrast between the 10 min and 60 min nap conditions.

This study was approved by the Institutional Review Board of the National University of Singapore and conducted according to the principles in the declaration of Helsinki. Participants were briefed about the study aims and procedures and provided written informed consent.

### Protocol

Participants completed all four conditions in a repeated-measures design: no nap, 10 min nap, 30 min nap, and 60 min nap. A balanced Latin square procedure was used to counterbalance the order of conditions and stimuli. Participants were not informed of the order of conditions or the nap condition they would undergo until arrival at the sleep laboratory at each visit.

Participants were instructed to keep habitual sleep habits throughout the experimental period (nocturnal sleep of 6–6.5 hr and sleep and wake times between 23:00 and 08:00, respectively) and to avoid napping on the day of each session. Participants wore an Actiwatch (Actiwatch 2, Philips Respironics Inc., Pittsburgh, PA) and filled in sleep diaries 3 days prior to each session to verify adherence with these instructions. Average TIB was 6.32 ± 0.48 hr and total sleep time (TST) was 5.23 ± 0.61 hr, durations that are typical in Singaporean young adults. Participants were asked to refrain from medication, nicotine, alcohol, caffeine, and vigorous exercise 24 hr prior to each session. They were instructed to have a normal-sized lunch by 11:30 before they arrived at the laboratory at 12:00. After their actigraphy and sleep diary records were verified, polysomnography was applied (including the wake group).

**Figure 1.**
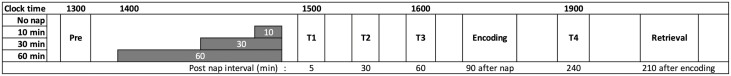
Study protocol. Participants completed all 4 conditions in a fully within design: no nap, 10 min nap, 30 min nap, and 60 min nap. The pre-nap test battery (Karolinska Sleepiness Scale, Digit Symbol Substitution Test, Positive and Negative Affect Scale, 3-min Psychomotor Vigilance Task) was administered at 1315. In the 10 min nap, 30 min nap, and 60 min nap conditions, lights-off times were 1420, 1400, and 1330, respectively. Post-nap tests were conducted at intervals of 5 min (to assess sleep inertia), 30 min, 60 min, and 240 min. The encoding session for the picture encoding task began 90 min after waking from the nap, and retrieval took place 210 min after encoding.

For each session, the pre-nap test was administered at approximately 13:15 (Figure 1). Given that participants were not sleep-restricted beyond their habitual amount of sleep, and based on previous nap experiments in our lab showing an average of 15.6 min sleep latency for an afternoon nap [26], we provisioned 30 min for participants to fall asleep. To reduce circadian influences on testing, we aimed to align the clock time of post-nap tests as closely as possible. In the wake group, participants were allowed to conduct their own activities in the lab under supervision. In the 10 min nap, 30 min nap, and 60 min nap conditions, lights-off times were 14:20, 14:00, and 13:30, respectively. Post-nap tests were conducted at 5 min (to assess sleep inertia), 30 min, 60 min, and 240 min upon waking from each of the 10 min, 30 min, and 60 min naps. As such, for all conditions, if sleep latency was 0 min, participants would be woken at 14:30 and their 5 min post-nap test would commence at 14:35. If sleep latency was maximal at 30 min, participants would be woken from their nap at 15:00 and the 5 min post-nap test would be at 15:05. Hence, deviations in the clock times of the post-nap tests did not exceed 30 min. The first test started at 1505 for the wake condition. The encoding session for the picture encoding task began 90 min after waking from the nap, and retrieval took place 210 min after encoding.

At the end of each session, participants were given a new sleep diary and provided with an Actiwatch for the next session which was scheduled at least 1 week later. At the end of the experiment, participants were debriefed and reimbursed accordingly.

### Polysomnography

Polysomnography was performed using six-channel EEG (Electroencephalography) montage (F3-A2, F4-A1, C3-A2, C4-A1, O1-A2, and O2-A1) according to the 10–20 system. Eye movement and muscle tone were recorded through left and right electrooculographic and submental electromyographic electrodes that are respectively referenced to A2 and A1. EEG, electrooculographic, and electromyographic signals were recorded using a Comet Portable EEG system from Grass Technologies (Astro-Med, Inc., West Warwick, RI). The sampling rate and the storage rate were 800 and 200 Hz, respectively. The low-pass and high-pass filters were set at 35 and 0.3 Hz for the EEG signals and 70 and 10 Hz for the electromyographic signals. Electrode impedance was kept below 5 kΩ.

Sleep was auto-scored in real-time in 30-sec epochs using the latest version of Z3Score cloud-based real-time autoscoring (https://z3score.com/) [27]. Sleep staging was manually verified by a trained technician following the American Academy of Sleep Medicine Manual for the Scoring of Sleep and Associated Events [28]. For all nap sessions, sleep commenced from the first indication of N1 sleep, following previous work [9]. The discrepancy in TST between auto-scored sleep and scoring by a trained technician was limited to ±10 min beyond which data were excluded from analysis.

The following sleep parameters were computed: TST, N1 latency (time from lights off to N1 onset), N2 latency, and durations of N1, N2, N3, and REM sleep.

### Picture encoding task

This task comprised an encoding session and a retrieval session 210 min later. Four versions of this task were created for each of the sessions and the order was counterbalanced for the nap conditions. In the encoding session, participants were instructed to look at each picture and depending on the picture set chosen for the session, determine using a key press (1—yes, 2—no) whether or not a specific feature (e.g. building/ no building, indoor/ outdoor, body of water/ no body of water, and street/ no street) was present in the picture. They were told to be as accurate as possible, and to view the pictures carefully as their memory for the pictures would be tested later. To control for the influence of differing strategy use, participants were advised with the same strategy—to try to remember the pictures by looking out for distinct objects in each scene. In the retrieval session, participants were tested for their recognition of the previously shown images which were intermixed with new images. Each picture was shown for 4 sec and participants responded with a key press (1—definitely did not see, 2—probably did not see, 3—unsure, 4—probably saw, and 5—definitely saw).

Responses were split into four outcome measures: (1) confidence ratings of four (probably saw) and five (definitely saw) to old images were classed as “hits,” (2) ratings of four and five to new images were “false positives,” (3) ratings of one (definitely did not see) and two (probably did not see) to old images were classed as “misses,” and (4) one and two ratings to new images were “correct rejections.” The non-parametric signal detection measure Aʹ [29] was calculated using hit rate (*H*) and false alarm rate (*F*) to account for participants’ response bias toward old or new responses with 0.5 indicating chance level performance [30, 31].


$$\begin{array}{l} A^{\prime }=.5+\left[sign\left(H-F\right)\frac{{\left(H-F\right)}^{2}+\left|H-F\right|}{4\max\left(H,F\right)-4HF}\right], \end{array}$$

where sign(H−F) equals +1 if H−F>0, 0 if *H* = *F*, and −1 otherwise, and max(H,F) equals either *H* or *F*, whichever is greater.

For each version of the task, 240 gray-scale pictures of identical luminance and dimensions were prepared, with half containing a specific feature (e.g. a building, a body of water) and a half without the feature (e.g. no building, no body of water). Each picture set was distinct from the others. In total, 160 of the pictures were used for the encoding session, and the retrieval session contained these 160 old pictures as well as 80 new distractor pictures.

### Cognitive test battery

The cognitive test battery consisted of two tasks and two questionnaires and was administered in the following order: Karolinska Sleepiness Scale (KSS) [32], Digit Symbol Substitution Test (DSST) [33], Positive and Negative Affect Scale (PANAS) [34], and a 3-min psychomotor vigilance task (PVT) [35]. The 3-min PVT was chosen to avoid exceeding the time allocation of the tests. The test battery took approximately 10 min to complete.

In the KSS, participants rated their level of self-reported sleepiness on a 9-point Likert scale (1—very alert, 9—very sleepy, and great effort to keep awake). The 2-min DSST was used as a measure of the speed of processing. In this task, participants were required to match symbols to digits as quickly as possible following a key shown on screen. The total number of correct trials was used as the critical measure. On the PANAS, participants responded to 20 adjectives describing positive and negative mood states on a 5-point Likert scale (1—very slightly, 5—extremely). Positive and negative affect scores are represented by the sum of the item responses. We also examined the “Alertness” and “Attentiveness” ratings on the PANAS. Finally, the 3-min PVT assessed levels of vigilance. At intervals varying randomly from 2000 ms to 10 000 ms, a counter on the screen appeared and participants were to press a key as quickly as possible. Lapses were defined as responses exceeding 500 ms.

### Statistical analyses

We compared participants’ nocturnal sleep prior to each session by including the within-participant factor of nap condition (wake, 10 min nap, 30 min nap, and 60 min nap) into a general linear model. A similar model was used to test differences in sleep architecture and baseline cognitive performance between nap conditions.

For the picture encoding task, we used a general linear model to test the significance of the difference in encoding accuracy as well as retrieval performance measured by Aʹ between nap conditions. Post hoc tests examined pairwise differences between the wake versus 10 min, 30 min, and 60 min nap conditions. For significant contrasts, we examined if memory differences compared to wake were due to nap-related changes in alertness by testing the significance of Pearson’s correlations between memory performance and KSS scores and PVT performance in the test battery assessed prior to the retrieval session. We also tested the contribution of sleep inertia by including markers of sleep inertia into the model and determining if significant effects remained (more details in the results section).

For the test battery assessing self-reported sleepiness, mood, speed of processing, and vigilance, we examined whether performance significantly differed across test intervals between each of the nap conditions. We included within-participant factors of nap condition (wake, 10 min nap, 30 min nap, and 60 min nap) and test interval (pre-nap, 5 min, 30 min, 60 min, and 240 min) into a general linear mixed model. An unstructured variance–covariance matrix was specified. Of central interest to the present study were the planned pair-wise contrasts which compared post-nap test performance relative to pre-nap between the wake group and each nap condition. While the main effects of nap length, test time, and their interactions are reported in Table 2, the planned contrasts are the focus of this investigation. Following Tassi and Muzet [23], we defined sleep inertia as a state of lower arousal and a decrement in performance occurring immediately after waking relative to a pre-sleep baseline. In our study, this would be observed by increases from baseline in self-reported sleepiness, poorer mood, or performance decrements at the 5 min post-nap test interval relative to that seen in the wake condition. Subsequently, the change from baseline on these metrics measured at 30 min, 60 min, and 240 min post-nap compared to the wake condition would allow us to evaluate the temporal evolution of benefits across nap lengths.

**Table 2.** Main effects of nap length, test interval, their interaction, as well as planned contrasts evaluating the change in self-reported sleepiness, mood, and performance from the pre-nap baseline test for each post-nap test interval for each of nap conditions

Outcome measure	*χ* ^2^	Planned contrasts				
		Condition (vs. no nap)	Time interval (vs. pre-nap)			
			5 min	30 min	60 min	240 min
			T			
KSS	(1) 13.08**	10 min	−2.07*	−2.88**	−3.46***	−2.22*
	(2) 203.14***	30 min	0.38	−1.25	−2.27*	−0.98
	(3) 31.06**	60 min	−2.69**	−3.82***	−3.78***	−3.34***
PANAS alert	(1) 28.22*** (2) 51.35*** (3) 35.47***	10 min	0.50	1.25	1.75	1.62
		30 min	−0.71	2.06*	2.98**	2.99**
		60 min	0.69	1.06	3.04**	3.66***
PANAS attentive	(1) 9.66* (2) 22.27*** (3) 27.76**	10 min	0.00	1.57	2.41*	1.35
		30 min	0.28	2.70**	2.83**	1.96
		60 min	0.84	3.19**	3.82***	3.23**
PANAS positive	(1) 12.87** (2) 39.11*** (3) 29.15**	10 min	0.93	2.69**	3.05**	1.93
		30 min	−0.18	2.25*	2.41*	2.07*
		60 min	1.11	3.01**	3.74***	3.62***
PANAS negative	(1) 3.01 (2) 55.55*** (3) 9.56	10 min	−1.45	−1.75	−2.00*	−1.07
		30 min	−0.44	−1.89	−1.91	−1.36
		60 min	0.07	−0.60	−0.82	−0.53
DSST accuracy	(1) 0.30 (2) 1.48 (3) 15.26	10 min	1.18	1.49	0.42	0.80
		30 min	0.58	1.17	1.54	0.72
		60 min	−1.76	0.66	−0.14	−0.27
DSST median RT	(1) 1.59 (2) 74.32*** (3) 42.08***	10 min	0.96	−0.53	−1.24	−1.91
		30 min	2.99**	0.16	−1.06	−1.31
		60 min	4.15***	0.20	0.14	−0.91
PVT lapses	(1) 18.09*** (2) 13.97** (3) 18.02	10 min	−1.60	−2.27*	−2.03*	−0.46
		30 min	−1.37	−2.96**	−2.25*	−2.30*
		60 min	−0.28	−1.71	−1.47	−1.39
PVT median RT	(1) 14.39** (2) 9.03 (3) 11.04	10 min	−1.02	−2.25*	−1.87	−1.14
		30 min	−0.32	−2.30*	−1.37	−1.10
		60 min	−0.08	−1.90	−1.53	−0.59

*p < 0.05, **p < 0.01, ***p < 0.001.

*χ* ^2^ values: Likelihood Ratio Test for (1) the main effect of nap length, (2) the main effect of test interval, and the (3) nap length (no nap, 10 min nap, 30 min nap, and 60 min) × test interval (pre-nap, 5 min, 30 min, 60 min, and 240 min) interaction.

*t* values: planned contrasts between wake and nap conditions for change in performance at each post-nap test interval (post-nap test interval—pre-nap baseline).

Bold values indicate statistically significant tests.

We followed up significant contrasts by performing Pearson’s correlations to examine associations between the sleep parameters (duration spent in N1, N2, N3, and REM) and performance on the particular outcome measure for the post-nap test. Given the number of and exploratory nature of the tests, we corrected for family-wise errors due to multiple comparisons using a Bonferroni correction.

## Results

### Nap sleep architecture

Participants’ average sleep latency did not significantly differ between the 10 min (10.28 ± 7.03 min, Table 1), 30 min (7.70 ± 4.86 min), and 60 min (7.90 ± 5.94 min) nap conditions (*p* = 0.16), and they spent an average time in bed of 24.53 ± 9.76 min, 43.82 ± 9.10 min, and 76.26 ± 10.57 min, respectively to achieve the required TST. Participants’ sleep efficiency was highest in the 60 min nap condition (82.43 ± 10.58 %), followed by the 30 min (74.70 ± 12.88 %) and 10 min nap (52.58 ± 15.95 %) conditions (10 min vs. 30 min, *p* < 0.001; 10 min vs. 60 min, *p* < 0.001).

The 10 min nap comprised mainly N1 (46.77 ± 28.06%) and N2 sleep (49.15 ± 26.40%) and contained significantly less N3 sleep compared to the 30 min (10 min nap, N3%: 2.57 ± 8.22; 30 min nap, N3%: 27.45 ± 22.63, *p* < 0.001) and 60 min naps (35.12 ± 21.16%, 10 min vs. 60 min, *p* < 0.001). The 30 min nap and 60 min nap did not differ in their percentage of N3 sleep (*p* = 0.27). There was no significant difference in the percentage of time spent in N2 between any of the naps (*p* = 0.06). Percentage of REM sleep was significantly higher in the 60 min condition (11.68 ± 14.26%) compared to the 10 min (1.50 ± 8.49%, *p* < 0.001) and 30 min conditions (0.83 ± 4.38%, *p* < 0.001).

For the 10 min nap condition, 18.8% woke from N1, 71.9% woke from N2, 6.3% woke from N3, and 3.1% woke from REM sleep. For the 30 min nap condition, 19.4% woke from N1, 25.8% woke from N2, 54.8% woke from N3, and 0% woke from REM sleep. For the 60 min nap condition, 20% woke from N1, 53.3% woke from N2, 6.7% woke from N3 and 20% woke from REM sleep.

### Memory encoding

**Figure 2.**
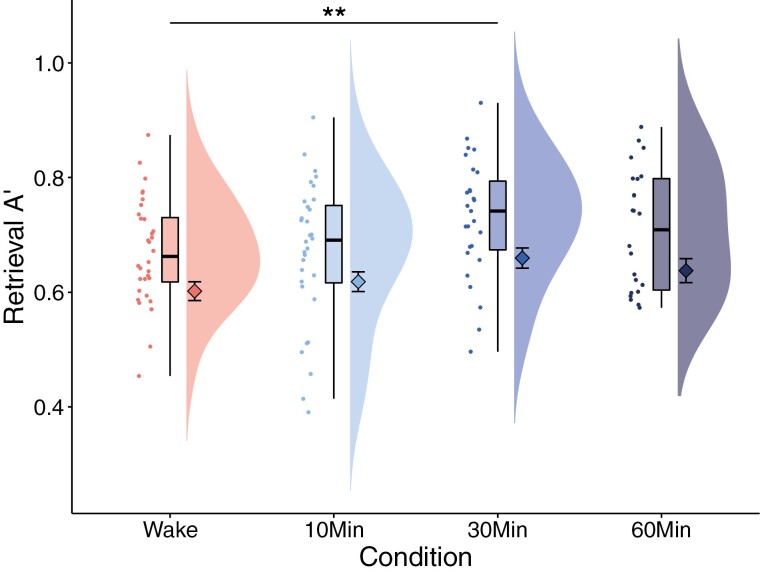
Retrieval performance (Aʹ) on the picture encoding task for the wake, 10 min, 30 min, and 60 min nap conditions. Diamonds with error bars represent the means and +/− standard error. **p < 0.01.

During the encoding phase, there was no significant difference between conditions in terms of accuracy of judgment (i.e. whether a specific feature was present, “yes” or “no,” mean accuracy >0.91, *χ*^2^ = 1.37, *p* = 0.71, Figure 2 and Table 3) or reaction times (mean RTs > 594.16 ms, *χ*^2^ = 3.52, *p* = 0.32).

**Table 3.** Mean (SD) for picture judgment accuracy and response times as well as memory performance measured by Aʹ

	*Encoding session*				Retrieval session	
	Judgment accuracy		RT (ms)		Aʹ	
Wake	0.91	(0.08)	653.50	(326.18)	0.67	(0.09)
10 min	0.94	(0.06)	646.08	(333.86)	0.67	(0.12)
30 min	0.92	(0.09)	594.16	(325.34)	0.73	(0.10)
60 min	0.92	(0.09)	661.85	(319.60)	0.71	(0.11)

At retrieval, there was a significant effect of nap condition for A’ (*χ*^2^ = 10.72, *p* = 0.01). Planned comparisons revealed that the 30 min nap, but not the 10 min, or 60 min naps, performed significantly better than the wake condition (30 min vs. wake: *t* = 2.85, *p* < 0.05, 10 min vs. wake: *t* = −0.03, *p* = 0.97, 60 min vs. wake, *t* = 1.35, *p* = 0.18, Figure 2). Neither PVT median RT nor KSS scores were significantly associated with A’ performance in the 30 min nap (*p*s > 0.71), suggesting that performance was not driven by improvements in vigilance or alertness.

Furthermore, we examined if sleep inertia may have contributed to the effect of nap duration on memory performance. As we observed significant sleep inertia on the DSST median RT measure (see the section below for details), we included this measure as covariate in the model. The main effect of nap condition remained significant, suggesting that differences in sleep inertia incurred after waking did not account for the superior memory performance in the 30 min nap.

No significant associations were observed between sleep parameters and memory retrieval performance for the 30 min nap condition (*p*s > 0.12).

### Self-reported sleepiness, alertness, and attentiveness

**Figure 3.**
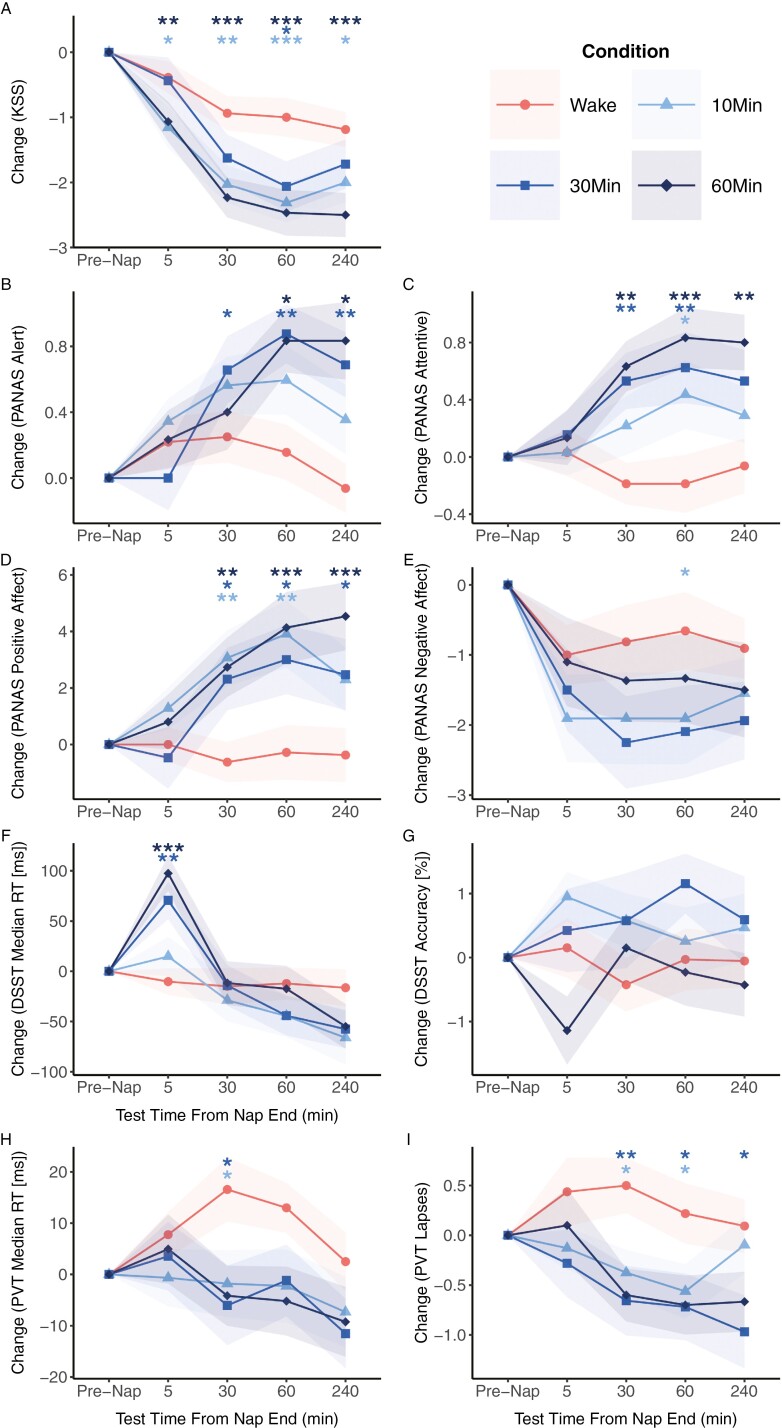
Post-nap test performance relative to pre-nap between the wake group and each nap condition was measured at 5 min, 30 min, 60 min, and 240 min intervals. Error bands represent standard errors.

There was no difference in self-reported sleepiness measured by KSS scores, PANAS alertness, and PANAS attentiveness ratings at the pre-nap baseline between nap conditions (*χ*^2^ > 3.98, *p* > 0.26). For the 10 min and 60 min naps, KSS scores were significantly reduced from 5 min post-nap up to 240 min post-nap (Table 2 and Figure 3). The 30 min nap showed a significant reduction in KSS scores at 60 min post-nap.

*p < 0.05, **p < 0.01, ***p < 0.001.

For the alertness measure on the PANAS, relative to wake, the 30 min, and 60 min naps led to significant increases in alertness scores emerging at 30 min and 60 min post-nap respectively that were sustained up to 240 min post-nap. Increases in alertness scores were not seen for the 10 min nap.

On the PANAS attentiveness scale, compared to wake, naps of all lengths increased attentiveness scores. For the 10 min nap, this was seen at 60 min post-nap. For the 30 min and 60 min naps, significant increases in attentiveness were seen from 30 min post-nap and lasted up to 60 min post-nap for the 30 min nap, and up to 240 min post-nap for the 60 min nap.

### Mood

At baseline, there were no significant differences between nap conditions in positive (*χ*^2^ = 2.83, *p* = 0.42) or negative affect scores (*χ*^2^ = 1.26, *p* = 0.74). No significant decreases in positive affect or increases in negative affect were seen 5 min post-nap (*p*s > 0.05, Table 2 and Figure 3). Subsequently, for positive affect, all nap durations showed significant increases compared to wake. Improvements in positive affect were sustained up to 240 min post-nap for the 30 min and 60 min nap, but only up to 60 min post-nap in the 10 min nap condition. No significant changes in negative affect from baseline compared to wake were observed in the 30 min and 60 min nap conditions (at all post-nap intervals, *p*s > 0.12). Only the 10 min nap showed a decrease in negative affect 60 min after waking (*p* < 0.05).

### Speed of processing

At the pre-nap baseline, there were no significant differences between nap conditions for DSST reaction times (*χ*^2^ = 0.72, *p* = 0.87) or accuracy (*χ*^2^ = 2.47, *p* = 0.48). Compared to the wake condition, both the 30 min and 60 min naps, but not the 10 min nap, showed a significant slowing in median response times on the DSST 5 min after waking (30 min nap: *p* < 0.05; 60 min nap: *p* < 0.001, 10 min nap: *p* = 0.89, Table 2 and Figure 3). However, by 30 min post-nap, response times returned to that of the wake condition (*p*s > 0.66) and were not significantly different from baseline compared to wake for the rest of the subsequent post-nap intervals (*p*s > 0.50). For all nap lengths, the change in DSST accuracy compared to pre-nap baseline did not differ significantly from wake for all post-nap intervals (*p*s > 0.14).

### Vigilance

At baseline, there were no significant differences between nap conditions for PVT median reaction times (*χ*^2^ = 1.01, *p* = 0.80) and lapses (*χ*^2^ = 2.61, *p* = 0.46). At 5 min after waking, relative to the wake condition, none of the nap conditions resulted in significantly longer median response times (*p*s > 0.53) or higher lapses on the PVT (*p*s > 0.13, Table 2 and Figure 3).

Subsequently, benefits emerged at 30 min post-nap for the 10 min and 30 min nap conditions which showed significantly improved response times compared to wake (*p*s < 0.05). However, these gains were no longer significant at 60 min and 240 min post-nap. The 60 min nap did not show improvements in response times compared to wake. For lapses, both the 10 min and the 30 min naps showed a significantly reduced number of lapses compared to the wake condition. This was sustained up to 240 min post-nap for the 30 min nap and up to 60 min for the 10 min nap.

### Correlations between nap parameters and test battery performance

For significant contrasts, we explored if sleep macrostructure parameters in the nap were associated with test performance at different test intervals. We found no significant correlations.

## Discussion

The present study adds to the existing literature on nap optimization for cognition by comparing 10 min, 30 min, and 60 min naps on their benefits to memory encoding, as well as extending test intervals to investigate self-reported and objective metrics of mood and performance. We found that all nap durations showed benefits for self-reported alertness and positive mood up to 240 min after napping. Only naps of 30 min significantly benefitted memory encoding compared to wake, and sleep inertia did not account for the differences in encoding performance. Factors that may have contributed to these effects, including extent of prior sleep deprivation and test delay interval, are discussed.

### Memory encoding

Most nap studies investigating effects on memory encoding have used longer naps of 90 min or more to make provision for greater amounts of NREM sleep [7]. This follows from earlier findings that napping may facilitate memory encoding through NREM-driven synaptic downscaling wherein memory encoding capacity is restored during the nap [36, 37]. However, not all studies have found associations with sleep parameters [4, 26, 38]. Here, we found that compared to wake, a 30 min nap significantly boosted encoding performance, and we did not find associations with sleep macrostructure. Although numerically A’ in the 30 min and 60 min nap conditions were close, compared to wake, encoding performance for the 60 min nap was not statistically significant.

In addition, significant effects of nap length on encoding performance remained after statistically controlling for sleep inertia, suggesting that sleep inertia did not explain the relatively better encoding performance in the 30 min nap. Moreover, within 30 min of waking, DSST reaction times returned to baseline levels for both the 30 min and 60 min naps, suggesting that sleep inertia had dissipated prior to encoding, which commenced 90 min after waking.

A boost in vigilance may have facilitated superior memory encoding as the 30 min nap was associated with vigilance improvement up to 240 min after waking. However, we did not observe vigilance improvement to be significantly correlated with encoding performance [30, 38, 39].

The length of nocturnal sleep in the night(s) prior to napping could also influence nap effects on memory encoding. Our previous work in adolescents using the same picture encoding task found that a longer 90 min nap following a night of sleep restriction significantly facilitated memory encoding [4, 38]. Unlike the present study where participants adhered to their habitual sleep schedule, the adolescents in our previous study received ~2 hr less than their usual amount of nocturnal sleep and ~3 hr shorter than the recommended amount for teens.

Differences may also arise depending on whether testing occurred on the same day or after intervening sleep. In our prior studies with adolescents, participants retrieved the pictures 2 days after recovery from sleep restriction. As such, the subsequent sleep opportunity may have conferred additional benefits to the encoded material [40]. Whether or not nap duration differences may be more prominent for tests occurring *the same evening* may be explored in future work.

### Benefits to sleepiness and positive mood

In comparison with objective measures, all nap durations alleviated self-reported sleepiness, and improved alertness and positive mood. Affective states influence work performance by impacting the willingness to dedicate attentional resources to a task [41], and also predict counter-productive work patterns or variability in work performance [42]. The elevated positive mood observed in the present study is also in line with our previous work showing that in sleep-restricted teens, 60–90 min naps improved positive mood but did not modify negative affect [43, 44]. Elsewhere, a study in a small sample of young adults also found that after a nap comprising 30 min TST, participants woke reporting increased levels of joy, while feelings of sadness were unchanged [45]. Following a 20 min nap, this pattern of increased happiness was also observed for habitual but not non-habitual nappers [18]. The non-uniform manner in which mood is impacted by sleep loss has been documented [46] and the present data suggests that improvements in mood following napping follow a similar pattern. Our findings shed light on the well-being benefits of naps, and future studies may examine how nap-related improvements in mood can have a direct impact on motivation and productivity at work.

Although previous studies have indicated a role for REM sleep in emotional regulation [47], here we found that despite the 60 min nap condition obtaining significantly more REM sleep than the 10 and 30 min conditions, improvements in positive mood compared to wake were not significantly different between nap conditions. This suggests that boosts in positive mood after napping may not be dependent on REM sleep, and are present even for short bouts of sleep with minimal REM.

### Vigilance

Improvements in objective measures of vigilance and speed of processing were less clear. While improvements in vigilance emerged half an hour after waking from the 10 min and 30 min naps, the 60 min nap did not show consistent benefits. As discussed, the less pronounced benefits to these domains compared to other studies may be related to participants keeping to their habitual short sleep schedule without deprivation beyond their usual amount of sleep. In support of this, none of the participants had more than two lapses or more than two false alarms for any of the PVT sessions, suggesting the presence of a ceiling effect. The 3-min PVT used here compared to the longer 10-min PVT may also have contributed to the decreased sensitivity of this task to nap effects under conditions of habitual sleep.

### Sleep inertia

Following Tassi and Muzet [23], we defined sleep inertia as the decrement in performance or self-reported state occurring immediately after waking (i.e. 5 min post-nap) relative to a pre-sleep baseline. We found no evidence of sleep inertia with the shortest 10 min nap. The 30 min nap was the only nap condition that did not show a significant decrease in KSS 5-min after waking. This may be attributed to the majority of participants (54.8%) waking from N3 sleep in the 30 min nap condition, whereas this occurred less in the 10 min (6.3%) and 60 min (6.7%) nap conditions. For the 30 min and 60 min naps, sleep inertia was also seen in the speed of processing. Numerically, compared to the 30 min nap, the 60 min nap had increased DSST reaction times compared to pre-nap. Although poorer DSST accuracy was also seen, this decrement was not statistically significant. The sensitivity of speed over accuracy as a marker of sleep inertia [48], particularly where severe sleep deprivation is absent [49–51], has been previously observed across tasks of varying complexity [25, 52].

Overall, we found minimal sleep inertia. For individuals on a habitual short sleep schedule, even with the hour-long nap, sleep inertia resolved within 30 min of waking suggesting that one may schedule cognitive work to begin ~30 min after waking from naps ranging from 10–60 min. Nonetheless, where severe sleep restriction or total sleep deprivation precedes the nap, it is likely that sleep inertia would be more pronounced regardless of task difficulty or domain [53].

### Limitations

In this study, we did not examine how habitual napping might impact nap benefits as we did not have sufficient statistical power to do so. Previous studies have shown differential benefits of naps depending on nap habit [25, 54], although this may be domain specific. Future studies may compare habitual and non-habitual nappers to investigate whether and how different nap durations may impact nappers differently. In general, for the number of questions addressed, our sample size may be considered small. Considering practical limitations, multi-center studies may be needed to resolve issues of statistical power.

The present study only examined a 210 min delay between encoding and retrieval on the picture encoding task. It is possible that benefits with the 60 min nap may have been observed if a longer delay was examined. Future work evaluating the relative efficacy of nap durations on memory encoding could extend delay intervals to investigate this possibility. Similarly, given that benefits of 30 min and 60 min naps were observed even up to 240 min post-nap, future studies could further extend test intervals to assess the temporal trajectory of benefits up to before bedtime.

Finally, as we did not examine nocturnal sleep following the nap, it remains unknown how naps of different durations would impact nocturnal sleep. However, previous experimental work in adolescents has found that naps of 60–90 min did not curtail nocturnal sleep [3, 54], and this is supported by observational studies showing that daytime napping in young and middle-aged adults does not impair sleep at night [55]. However, the relationship between napping and nocturnal sleep may be more mixed in older adults depending on baseline sleep health and total sleep obtained across a 24-hr period [56–58], highlighting the need to experimentally evaluate the efficacy of nap durations across different age groups.

## Conclusions

All nap durations improved alertness and increased positive mood. However, compared to wake, a 30 min nap incurred minimal sleep inertia and benefitted memory encoding. With 10–15 min of sleep latency in mind, one should apportion ~40–45 min to obtain 30 min of sleep for a midday refresh for learning and mood improvement.

## Data Availability

The data underlying this article are available upon reasonable request.
